# An Important Role of α-Hemolysin in Extracellular Vesicles on the Development of Atopic Dermatitis Induced by *Staphylococcus aureus*

**DOI:** 10.1371/journal.pone.0100499

**Published:** 2014-07-03

**Authors:** Sung-Wook Hong, Eun-Byul Choi, Taek-Ki Min, Ji-Hyun Kim, Min-Hye Kim, Seong Gyu Jeon, Byung-Jae Lee, Yong Song Gho, Young-Koo Jee, Bok-Yang Pyun, Yoon-Keun Kim

**Affiliations:** 1 Department of Life Sciences, Pohang University of Science and Technology (POSTECH), Pohang, Republic of Korea; 2 Department of Pediatrics, Sooncheonhyang University College of Medicine, Seoul, Republic of Korea; 3 Department of Medicine and Institute of Convergence Medicine, Ewha Womans Medical Center, Seoul, Republic of Korea; 4 Department of Allergy and Clinical Immunology, Samsung Medical Center, Sungkyunkwan University School of Medicine, Seoul, Republic of Korea; 5 Department of Internal Medicine, Dankook University College of Medicine, Cheonan, Republic of Korea; University of Liverpool, United Kingdom

## Abstract

Skin barrier disruption and dermal inflammation are key phenotypes of atopic dermatitis (AD). *Staphylococcus aureus* secretes extracellular vesicles (EVs), which are involved in AD pathogenesis. Here, we evaluated the role of EVs-associated α-hemolysin derived from *S. aureus* in AD pathogenesis. α-hemolysin production from *S. aureus* was detected using western blot analyses. The cytotoxic activity of α-hemolysin on HaCaT keratinocytes was evaluated by measuring cell viability after treating cells with soluble and EVs-associated α-hemolysin. To determine the type of cell death, HaCaT keratinocytes were stained with annexin V and 7-AAD. The *in vivo* effects of α-hemolysin were evaluated by application of soluble and EV-associated α-hemolysin on the mouse skin. The present study showed that increased α-hemolysin was produced by *S. aureus* colonized on AD patients compared to healthy subjects. α-hemolysin production was also related to AD severity. In addition, EV-associated α-hemolysin was more cytotoxic to HaCaT keratinocytes than soluble α-hemolysin, and α-hemolysin-negative EVs did not induce keratinocyte death. EV-associated α-hemolysin induced necrosis, but soluble α-hemolysin induced apoptosis of keratinocytes. *In vivo,* skin barrier disruption and epidermal hyperplasia were induced by soluble and EV-associated α-hemolysin. However, AD-like dermal inflammation was only caused by EV-associated α-hemolysin. Moreover, neither skin barrier disruption nor AD-like skin inflammation was induced by α-hemolysin-negative EVs. Taken together, α-Hemolysin secreted from *S. aureus*, particularly the EV-associated form, induces both skin barrier disruption and AD-like skin inflammation, suggesting that EV-associated α-hemolysin is a novel diagnostic and therapeutic target for the control of AD.

## Introduction

Atopic dermatitis (AD) is a chronic inflammatory skin disease that is characterized by eczematous lesions with pruritus and xerosis [1]. AD skin lesions show distinct features, such as disrupted barrier function with epidermal hyperplasia and *Staphylococcus aureus* colonization. In particular, abnormal skin barrier function induced by the death of keratinocytes is one of the major causes in the etiology of AD [2], [3], [4]. Through the disrupted skin barrier, pathogen-associated antigens and allergens can penetrate the skin and subsequently affect host immune responses. Skin lesions of AD patients show increased keratinocyte cell death induced by immunologic mediators [5], [6], [7].

*S. aureus* is a gram-positive bacterium and notorious pathogen that can induce many human diseases [8]. Human skin and nasal anterior are well-known reservoirs of *S. aureus,* and it has been reported that carriage of *S. aureus* is related to allergic diseases. In particular, *S. aureus* is closely related to AD because it colonizes the skin lesions of most AD patients and augments disease severity [9], [10]. *S. aureus* affects the host immune system by producing pathogenic molecules and toxins. For example, *S. aureus* produces staphylococcal enterotoxins that can induce excessive T-cell responses, as well as hemolysins, that cause cell death by forming heptameric membrane pores [8], [11]. Among these, α-hemolysin (also called as α-toxin) is an important toxin, which kills many types of cells [8], [12], [13]. It has also been reported that α-hemolysin can target keratinocytes and is related to AD disease severity [14], [15].

Recent evidence indicates that *S. aureus* secretes extracellular vesicles (EVs) as well as soluble toxins [16]. EVs derived from *S. aureus* are 20-200-nm vesicular structures that are membrane-enveloped spherical complexes and contain many proteins, DNA, RNA, and toxins. *S. aureus* EVs show potent immunogenicity and are related to AD pathogenesis [17]. Proteome analyses showed that EVs harbor pathogenic toxins, including α-hemolysin [16]. Thus, we hypothesized that α-hemolysin, particularly the EV-associated form, is a key mediator of AD pathogenesis.

## Results

### Comparison of α-hemolysin production between AD patients and healthy subjects

**Figure 1:**
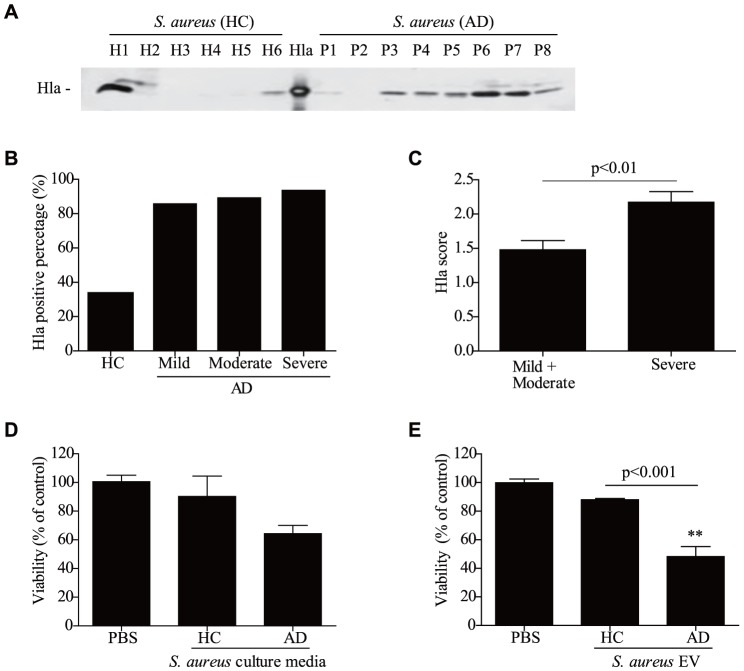
*S. aureus* on atopic dermatitis skin produces α-hemolysin. **A,** Detection of α-hemolysin in culture media of *S. aureus* isolated from the skin of healthy controls (HC) and atopic dermatitis patients (AD). **B,** The percentage of α-hemolysin-producing *S. aureus* from 90 AD patients. **C,** The amount of α-hemolysin in culture media was evaluated by scoring western blot band sizes from 0 to 3. **D** and **E,** Human keratinocyte viability after treatment with 10 µg/ml *S. aureus* culture media (D) and 25 µg/ml EVs (E) for 24 hr. ** P<0.01 versus the PBS group.

To evaluate the relationship between α-hemolysin production from *S. aureus* and AD, α-hemolysin levels were measured in culture media of *S. aureus* from AD patients and healthy controls. All bacteria were cultured under the same conditions and α-hemolysin levels in the culture media were measured by western blot. Although two of six samples of *S. aureus* from healthy controls produced α-hemolysin, most *S. aureus* from AD patients (seven of eight) produced it (**Fig. 1** **, A**). Furthermore, when α-hemolysin production was measured in *S. aureus* culture media from 90 AD patients, 91% of *S. aureus* from AD patients were positive for α-hemolysin compared to 33% of healthy controls (**Fig. 1** **, B and** **Table 1**). In terms of α-hemolysin production according to AD severity, α-hemolysin production was significantly higher from *S. aureus* from the severe group compared to *S. aureus* from the mild and moderate groups (**Fig. 1** **, C and** **Table 1**). These findings suggest that α-hemolysin from *S. aureus* is related to AD disease development and/or progression.

**Table 1:** Demographic and clinical characteristics of atopic dermatitis patients.

		Mild	Moderate	Severe	Total
		n = 7	n = 37	n = 46	n = 90
Age, months		66.43	51	62	55
(range)		(36–145)	(4–163)	(2–510)	(2–510)
Sex (male: female)		01:02.5	01:01.2	1:01	01:01.1
MRSA (%)		42.8	43.2	28.6	38.9
α-hemolysin (%)¶	0	14.3	10.8	6.5	8.9
	1	28.6	48.7	26.1	35.5
	2	42.8	24.3	10.9	18.9
	3	14.3	16.2	56.5	36.7

Values shown are age in months (median), ratio of males versus females, percentage of methicillin-resistant *Staphylococcus aureus*, and percentage of patients colonized with α-hemolysin-producing *S. aureus*.

¶ The percentage was determined by the amount of α-hemolysin produced by *S. aureus* isolated from the patients. The amount of α-hemolysin was measured by band intensity using Multi Gauge V3.1. Scores are as follows: 0: zero; 1: up to 6000 arbitrary units (AU); 2: from 6001 to 15,000 AU; and 3: over 15,001 AU.

### The effect of α-hemolysin on keratinocyte cell death

Numerous reports have shown that α-hemolysin can kill many types of cells, including epidermal keratinocytes [12], [13], [14]. Because α-hemolysin-producing *S. aureus* were more frequent in AD patients compared to healthy controls, we evaluated the effect of α-hemolysin on keratinocyte death by measuring cell viability after treatment with bacterial culture media. We found that HaCaT keratinocyte viability was decreased upon treatment with culture media of *S. aureus* from AD patients compared to healthy controls (**Fig. 1** **, D**). In addition, we evaluated the effect of *S. aureus* EVs on HaCaT keratinocyte death. The results indicate that keratinocyte viability was significantly decreased upon treatment with *S. aureus* EVs from AD patients (**Fig. 1** **, E**). These findings suggest that α-hemolysin in *S. aureus* EVs may be an important etiologic agent in AD pathogenesis.

### The role of α-hemolysin in *S. aureus* EVs on keratinocyte cell death

**Figure 2:**
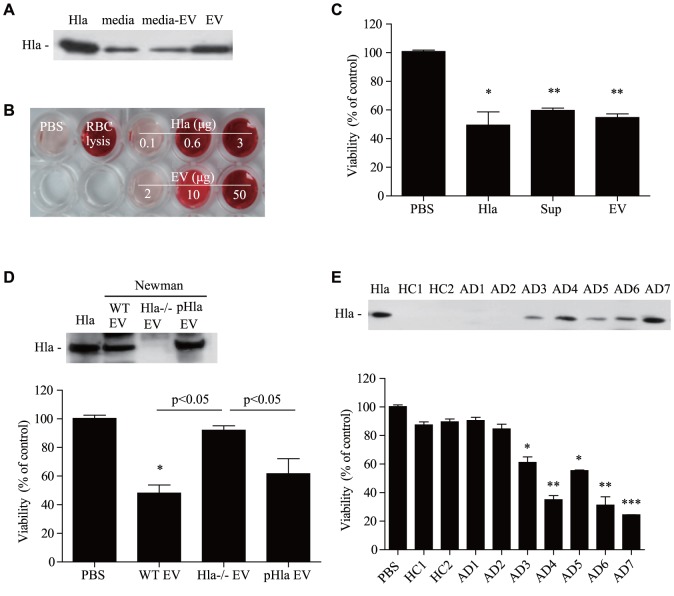
α-Hemolysin in *S. aureus* EVs is a key factor for EVs cytotoxicity. **A,** The presence of α-hemolysin in culture media, EVs-removed culture media (media-EVs), and EVs from the *S. aureus* ATCC14458 strain. **B,** Hemolytic function of soluble α-hemolysin and EVs. **C,** Viability of human keratinocytes after treatment with soluble hemolysin (5 µg/ml), *S. aureus* culture media (10 µg/ml), and EVs (20 µg/ml). **D,** α-Hemolysin in EVs from the Newman strain, α-hemolysin-deficient mutant strain, and α-hemolysin complemented strain (pHla). Human keratinocyte viability after treatment with each EVs (40 µg/ml). **E,** α-Hemolysin in EVs from randomly selected *S. aureus* from healthy controls (HC) and atopic dermatitis (AD) patients. Viability of human keratinocytes after treatment with EVs (25 µg/ml). * P<0.05; ** P<0.01 versus the PBS group.

Based on these data, we sought to determine whether *S. aureus* EVs harbor α-hemolysin, which induces keratinocyte cell death. For these experiments, we used the *S. aureus* 14458 strain, a reference strain used previously [16], [17]. Our data show that α-hemolysin was present in both culture media and EVs from the *S. aureus* 14458 strain (**Fig. 2** **, A**). In terms of α-hemolysin hemolytic activity, we found that hemolysis was induced by *S. aureus* EVs in a dose-dependent manner, as well as by a soluble form of α-hemolysin (sHla) and the *S. aureus* culture media (**Fig. 2** **, B and Fig. S1, A**). Next, to evaluate the cytotoxic effect of α-hemolysin on keratinocytes, *S. aureus* EVs were added to HaCaT keratinocytes. Keratinocyte viability was significantly decreased upon treatment with *S. aureus* EVs, *S. aureus* culture media, and sHla compared to PBS alone (**Fig. 2** **, C**). *S. aureus* EVs and sHla also killed primary human keratinocytes (data not shown). To elucidate the role of α-hemolysin in EVs on keratinocyte cell death, we performed experiments using *S. aureus* strains that produce various amounts of α-hemolysin. We found that the amounts of α-hemolysin in *S. aureus* EVs were positively associated with keratinocyte death (**Fig. S1, B**). Next, to evaluate the effect of α-hemolysin deficiency on keratinocyte death, we isolated EVs from α-hemolysin-positive WT (Newman strain) and α-hemolysin-deficient mutant strains. Cell viability of HaCaT keratinocytes was significantly higher after treatment with *S. aureus* EVs from the α-hemolysin-positive strain compared to the α-hemolysin-negative strain. In addition, keratinocyte death was reversed by treatment with EVs isolated from the α-hemolysin-negative strain that complemented α-hemolysin using a plasmid (**Fig. 2** **, D**). Furthermore, the results indicate that EVs from α-hemolysin-negative *S. aureus* strains from either AD patients or healthy controls did not induce HaCaT keratinocyte death, whereas death was induced by α-hemolysin-positive *S. aureus* strains from AD patients (**Fig. 2** **, E**). Taken together, these findings suggest that α-hemolysin in *S. aureus* EVs are a key player in AD pathogenesis via keratinocyte death.

### Comparison between soluble and EV-associated α-hemolysin on keratinocyte cell death

**Figure 3:**
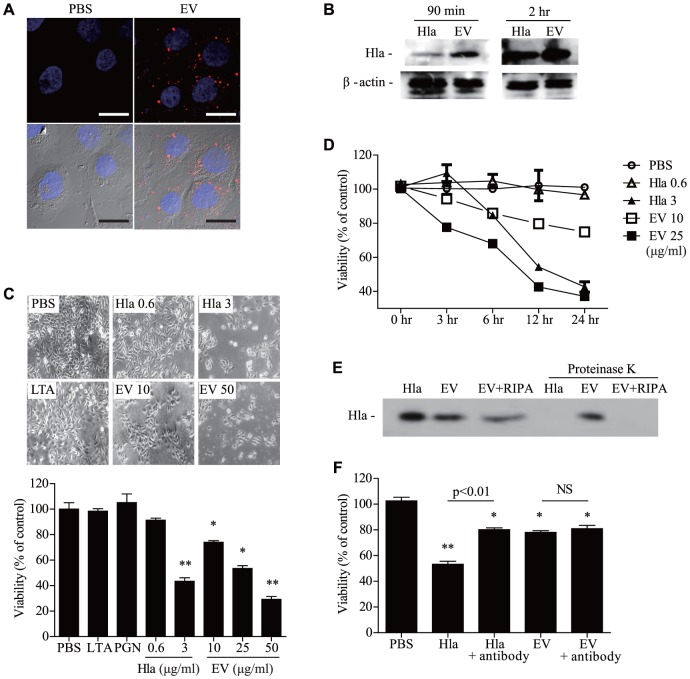
EVs are more potent mediators of keratinocyte death compared to soluble α-hemolysin. **A,** Confocal microscopy of human keratinocytes with DiI-labeled *S. aureus* EVs (red: *S. aureus* EVs, blue: nucleus). *S. aureus* EVs and nucleus are shown merged on DIC image (lower panel). Scale bar, 20 µm. **B,** α-Hemolysin in keratinocytes after treatment with identical amounts of soluble α-hemolysin and EVs. **C,** Viability of keratinocytes after treatment with each reagent. **D,** Time dependence of cell death. **E,** α-Hemolysin on intact and disrupted EVs after treatment with proteinase K. **F,** Keratinocyte viability after treatment with soluble α-hemolysin (3 µg/ml) and EVs (10 µg/ml) with the anti-α-hemolysin antibody (5% of culture media volume). * P<0.05; ** P<0.01 versus the PBS group; NS, not significant.

Although sHla was reported to act on the plasma membrane of keratinocytes [14], the working mechanism of *S. aureus* EVs on keratinocyte death remains unknown. First, we evaluated the cellular localization of EVs. When fluorescence-labeled EVs were added to HaCaT keratinocytes, EVs were internalized into the cytoplasm (**Fig. 3** **, A**). In our measurements, EVs contained approximately 0.6 µg of α-hemolysin after treatment with 10 µg of EVs (quantified by total protein amount) (**Fig. S2**). When HaCaT keratinocytes were treated with equal amounts of soluble and EVs forms of α-hemolysin, α-hemolysin in EVs was more effectively delivered into the keratinocytes (**Fig. 3** **, B**). Furthermore, to compare the cytotoxic effects of EV-associated α-hemolysin and sHla, HaCaT keratinocytes were treated with equal amounts of α-hemolysin in the soluble and EV forms. This study showed that cytotoxicity was enhanced after treatment with EV-associated α-hemolysin compared to sHla (**Fig. 3** **, C**). Keratinocyte death was induced faster by EV-associated α-hemolysin versus sHla (**Fig. 3** **, D**). Together, these findings suggest that compared to sHla, EV-associated α-hemolysin potently induces keratinocyte death.

### α-hemolysin localization in *S. aureus* EVs

Generally, secreted soluble toxins are neutralized and lose their activity by engaging the host immune system [18], [19], [20]. Compared to soluble toxins, EVs may protect toxins by enveloping them with cell membrane. Our data show that EV-associated α-hemolysin remained intact after proteinase K treatment; however, after EVs were disrupted by lysis buffer, EV-associated α-hemolysin was degraded by proteinase K (**Fig. 3** **, E**). This finding suggests that α-hemolysin is localized in the EV lumen, not on the EV surface. Moreover, when both soluble and EV-associated α-hemolysin were treated with the anti-α-hemolysin antibody, keratinocyte cell death induced by EV-associated α-hemolysin was unaffected, whereas the cytotoxicity induced by sHla was reversed (**Fig. 3** **, F**). Collectively, these findings suggest that α-hemolysin in the EV lumen enhances killing of keratinocytes and evasion of host immune defenses.

### Comparison of cell death mechanisms between EV-associated and soluble α-hemolysin

**Figure 4:**
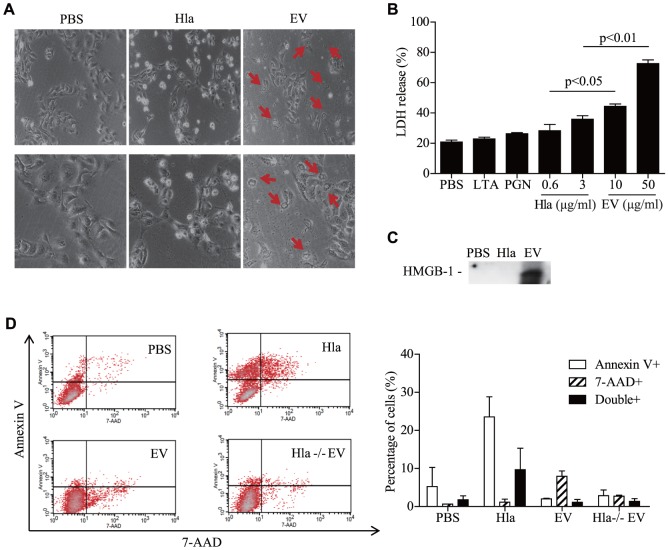
*S. aureus* EVs induces necrotic cell death, in contrast to soluble α-hemolysin. **A,** Micrographs of cell death by soluble α-hemolysin (3 µg/ml) and EVs (25 µg/ml). Pictures were taken under microscope at x 100 (upper panel) and x 200 (lower panel) magnification. Red arrows indicate ruptured cells. **B,** LDH levels in culture media after treatment with soluble α-hemolysin and EVs. **C,** HMGB-1 in culture media after treatment with soluble α-hemolysin and EVs. **D,** Flow cytometry analyses of annexin V and 7-AAD staining.

Although several reports have suggested that sHla induces host cell apoptosis [12], [21], [22], the exact mechanism of EV-associated α-hemolysin in keratinocyte death is unknown. We found that the morphology of cell death differed between soluble and EV-associated α-hemolysin; HaCaT keratinocytes were rounded and many cells were floating upon sHla treatment, which is indicative of apoptotic cell death; whereas cells underwent cell rupture upon EVs treatment, suggestive of necrosis (**Fig. 4** **, A**). In accordance with the observed cell morphology, LDH release, a marker of necrosis or cell rupture, was significantly increased after EVs treatment (**Fig. 4** **, B**). In addition, high-mobility group box (HMGB)-1, another marker for necrosis [23] was detected in culture media of EV-treated HaCaT keratinocytes, but not from that of sHla-treated cells (**Fig. 4** **, C**). Flow cytometry also showed that the number of 7-AAD-positive cells (necrotic cells) increased after EVs treatment, whereas annexin V-positive cells (apoptotic cells) increased after sHla treatment (**Fig. 4** **, D**). To sum up, these findings suggest that EV-associated α-hemolysin induces necrotic cell death by carrying α-hemolysin into the cytoplasm of keratinocytes, whereas sHla induces keratinocyte death via apoptosis.

### *In vivo* effects of EV-associated and soluble α-hemolysin on skin barrier disruption

**Figure 5:**
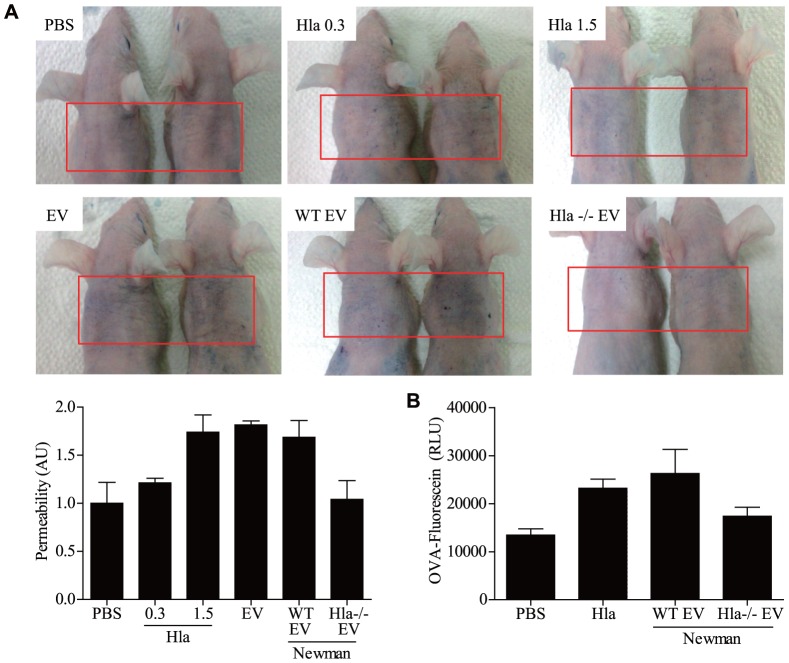
α-Hemolysin from *S. aureus* induces skin barrier disruption. **A,** Evans blue dye penetration into mouse skin after treatment with soluble α-hemolysin, EVs (5 µg), EVs from the Newman strain (10 µg), and EVs from the α-hemolysin-deficient strain (10 µg) (n = 2 mice per group). **B,** Level of penetrated fluorescein-labeled OVA in skin treated with soluble α-hemolysin (1.5 µg), Newman EVs (10 µg), and α-hemolysin-deficient EVs (10 µg) (n = 3 mice per group).

Because keratinocytes are major constituents of the skin barrier, keratinocyte death is a key contributor of skin barrier disruption in AD pathogenesis [2], [5]. To examine the *in vivo* effects of EV-associated α-hemolysin on skin barrier disruption, EVs from α-hemolysin-positive and -negative *S. aureus* strains, as well as sHla, were applied to the back skin of mice. Evans blue dye penetration was enhanced by α-hemolysin-positive EVs or sHla treatment compared to PBS or α-hemolysin-negative EVs treatment (**Fig. 5** **, A**). Next, we aimed to evaluate the *in vivo* effects of skin barrier disruption on the penetration of high-molecular-weight allergens. Therefore, fluorescein-labeled ovalbumin (OVA) was administered to EV- and sHla-treated skin, and OVA levels were measured in excised skins by quantification of fluorescein. We found that OVA-fluorescein levels were increased in mice treated with α-hemolysin-positive EVs or sHla compared to α-hemolysin-negative EVs or PBS treatment (**Fig. 5** **, B**). Together, these findings suggest that both EV-associated and soluble α-hemolysin induce skin barrier disruption via keratinocyte cell death and consequently enhance penetration of high-molecular-weight allergens.

### Effects of EV-associated and soluble α-hemolysin on pro-inflammatory mediator production by keratinocytes

**Figure 6:**
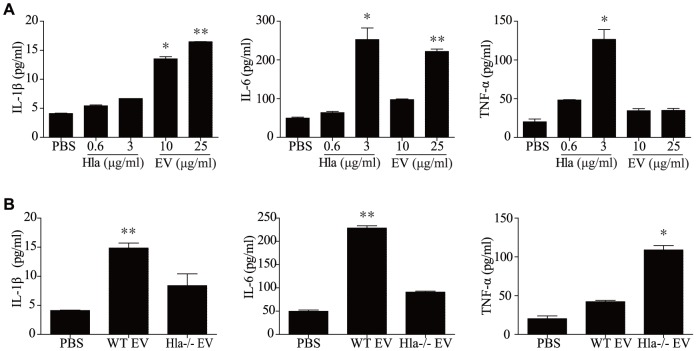
*S. aureus* EVs and soluble α-hemolysin induce production of different cytokines. **A** and **B,** Pro-inflammatory cytokines in human keratinocyte culture media after treatment with soluble α-hemolysin and EVs (A) and 40 µg/ml of EVs from each strain (B). * P<0.05; ** P<0.01 versus the PBS group.

Our previous reports showed that *S. aureus* EVs induced pro-inflammatory cytokine production by dermal fibroblasts and airway epithelial cells [17], [24]. To evaluate the effect of EV-associated and soluble α-hemolysin on the production of pro-inflammatory mediators from keratinocytes, we measured the production of pro-inflammatory cytokines from HaCaT keratinocytes after treatment with equal amounts of α-hemolysin in the EV and soluble forms. We found that the cytokine production profile differed between EV-associated and soluble α-hemolysin. IL-6 was enhanced by both EV-associated and soluble α-hemolysin, IL-1β was enhanced only by EV-associated α-hemolysin, and TNF-α was enhanced only by sHla (**Fig. 6** **, A**). Moreover, IL-1β and IL-6 production by keratinocytes was decreased after treatment with EVs derived from the α-hemolysin-negative *S. aureus* strain compared to the α-hemolysin-positive strain, whereas TNF-α production was enhanced in the former group versus the latter group (**Fig. 6** **, B**). Collectively, these findings suggest that EV-associated α-hemolysin induces IL-1β and IL-6 production from keratinocytes but may inhibit TNF-α production induced by *S. aureus* EVs or other signals.

### Effects of EV-associated and soluble α-hemolysin on the development of AD-like skin inflammation

**Figure 7:**
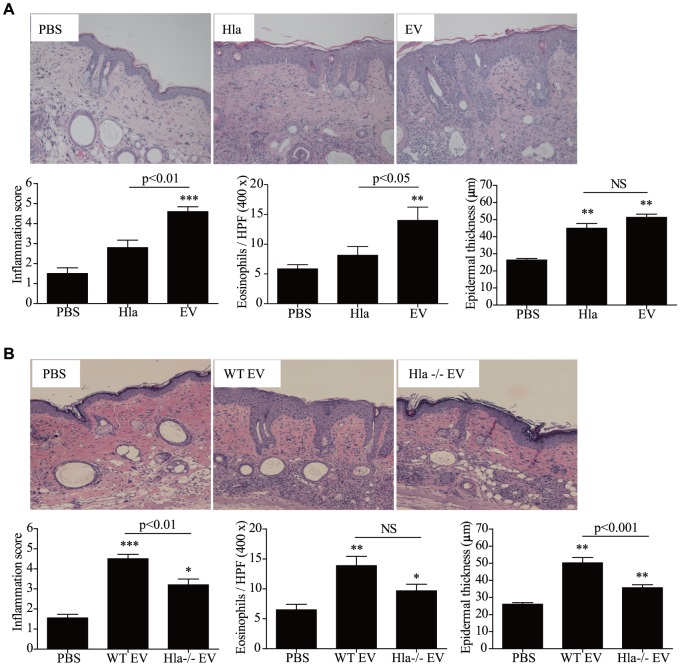
α-Hemolysin-positive *S. aureus* EVs induces atopic dermatitis-like skin inflammation. **A** and **B,** Skin alterations after treatment with 5 µg of soluble α-hemolysin and EVs (A) and 10 µg of EVs from the Newman wild-type and α-hemolysin-deficient strains (B) (n = 5 mice per group). * P<0.05; ** P<0.01; *** P<0.001 versus the PBS group; NS, not significant.

Finally, we evaluated *in vivo* effects of EV-associated and soluble α-hemolysin on the development of skin inflammation. To do this, the same amounts of α-hemolysin in the EV-associated and soluble forms were administered epicutaneously into the mouse skin and histological alterations were evaluated. Dermal infiltration of inflammatory cells, particularly eosinophils, was enhanced by EV-associated α-hemolysin but not by sHla. However, both forms of α-hemolysin increased epidermal cell hyperplasia (**Fig. 7** **, A**). Furthermore, dermal infiltration of inflammatory cells, including eosinophils, and epidermal thickening were reduced in skin treated with repeated applications of α-hemolysin-negative EVs (**Fig. 7** **, B**). Taken together, our data show that α-hemolysin in *S. aureus* EVs are a key player for the development of AD phenotypes, including epidermal thickening and eosinophilic inflammation in the dermis.

## Discussion

If the skin barrier is disrupted, pathogen-associated antigens and allergens can penetrate into the human body. Skin barrier disruption is considered one of the major causes of AD exacerbation, but many studies have focused on intrinsic factors, such as host molecules that maintain the skin barrier [4], [25]. Though it is known that staphylococcal toxins can affect skin barrier integrity for a long time, the role of α-hemolysin in skin barrier disruption via killing keratinocytes have been reported recently [26], [27]. In the present study, we elucidated the role of extrinsic factor (α-hemolysin and EVs) on the development of skin barrier disruption and AD-like inflammation. The present data show that α-hemolysin-producing *S. aureus* had colonized AD patients, and that soluble and EV-associated α-hemolysin induce keratinocyte cell death, consequently enhancing skin penetration of high-molecular-weight allergens. However, EV-associated α-hemolysin was found to induce keratinocyte necrosis, whereas sHla induced keratinocyte apoptosis. Additionally, EV-associated α-hemolysin induced epidermal thickening and eosinophilic inflammation in the dermis, whereas the soluble form induced only epidermal thickening. This is the first report that α-hemolysin in EVs derived from *S. aureus* induces skin barrier disruption and AD-like skin inflammation, predominantly via keratinocyte necrosis and the production of pro-inflammatory mediators by keratinocytes.

*S. aureus* secretes toxins, including α-hemolysin, as both soluble and EV-associated forms. EV-associated toxins have some advantages in intercellular communication compared to soluble forms. Toxins in the EV lumen, such as EV-associated α-hemolysin, can be protected from clearance by host defense systems, including antibody-mediated neutralization and protease-mediated destruction, enabling toxins to retain their function and travel long distances without interference [28]. In addition, because EVs are membrane-enveloped complexes, they can deliver their contents easily into the cytoplasm by fusing with the host cell membrane or by endocytosis after interaction between ligands on EVs and receptors on host cells [18], [29]. Indeed, the present data show that *S. aureus* EVs were internalized into the cytoplasm of keratinocytes and that EVs efficiently delivered α-hemolysin to the cytoplasm. Collectively, these findings suggest that EVs-associated toxins are key molecules in disease pathogenesis.

EVs derived from several bacteria kill host cells by transferring cytotoxic factors [29], [30], [31]. It was reported that *S. aureus* EVs can induce death of human epidermoid cancer cells [32]. Additionally, recent data indicate that α-hemolysin associated with *S. aureus* EVs can induce death of cervical cancer cells [33]. In addition, the present data showed that EV-associated α-hemolysin can also induce keratinocyte necrosis, whereas the soluble form induces apoptosis. This difference can be partly attributed to the differences in delivery efficacy between EV-associated and soluble α-hemolysin. We can speculate that α-hemolysin can be efficiently delivered by EVs and that a high α-hemolysin concentration in the cytoplasm attacks cell membranes and/or cytoplasm organelles, thereby initiating necrosis pathways.

Cell death via necrosis or apoptosis can result in immunologically distinct consequences [22]. The present data showed that EV-associated α-hemolysin up-regulateed the production of IL-6, a key mediator of Th17 polarization, which is associated with keratinocyte necrosis. In contrast, sHla enhanced the production of TNF-α, but not of IL-6. Our previous data demonstrated that skin exposure to *S. aureus* EVs induced a Th17-cell response in regional lymph nodes and ultimately resulted in AD-like skin inflammation [17]. Together, these findings suggest that *S. aureus* EVs induce skin barrier disruption and Th17-associated inflammation in the dermis via effective delivery of α-hemolysin into keratinocytes.

The expression of toxins from *S. aureus* can be regulated by environmental stress, and also host factors, such as filaggrin production [11], [34], . In the present study, most of *S. aureus* isolated from the skin of AD patients was found to produce α-hemolysin, as soluble and EV-associated forms, which can induce skin barrier disruption. In contrast, we can hardly detect the production of α-hemolysin from *S. aureus* isolated from healthy control subjects. Moreover, the levels of α-hemolysin in lavage fluids from the AD patient skin were found to be positively correlated with AD severity. Because EVs are produced via shedding of bacterial membrane and contain many pathogenic molecules [16], [18], it is hard to define the effect of EVs by deleting or adding specific molecules. We can assume that many proteins interact with other proteins and the relationship between various proteins can affect overall characteristics of EVs. Nevertheless, out present findings suggest that α-hemolysin, especially EV-associated form, is a good biomarker for the diagnosis and therapy of AD.

In summary, the present study showed that α-hemolysin, present in the EV lumen, induces skin barrier disruption and AD-like skin inflammation via keratinocyte necrosis and/or up-regulation of pro-inflammatory mediator production from keratinocytes. Moreover, *S. aureus* colonized on AD patient skin secretes α-hemolysin, which is significantly related to AD severity. These findings indicate that α-hemolysin, particularly the EV-associated form, is a novel target for diagnosis and treatment of AD.

## Materials and Methods

### Ethics statement

This study was carried out in strict accordance with the recommendations in the Guide for the Care and Use of Laboratory Animals of the National Institute of Health. The experimental protocols were approved by the Institutional Animal Care and Use Committee at POSTECH, Pohang, Republic of Korea (Permit Number: 2011-01-0027). All animal experiments were planned in order to minimize mice suffering. The study protocol for human samples was approved by the Ethics Committee of Seoul Suncheonhyang Hospital (Permit Number: 2010-01-0016). Participants provided their written informed consent to participate in the present study.

### Mice

SKH-HR1 hairless mice were purchased from Charles River Laboratories Japan, Inc. (Yokohama, Japan) and were bred in a specific-pathogen-free facility at Pohang University of Science and Technology (POSTECH; Pohang, Republic of Korea).

### Keratinocytes and bacteria

Immortalized human epidermal keratinocytes (HaCaT cells) were kindly donated by Jeung-Hoon Lee (Chungnam National University, Daejeon, Korea). Primary human epidermal keratinocytes (HEK cells) were purchased from ScienCell (Carlsbad, CA). HaCaT cells were maintained in DMEM (Hyclone Laboratories, South Logan, UT) containing fetal bovine serum (Gibco) and antibiotics (Hyclone Laboratories). ATCC14458 *S. aureus* strain was purchased from ATCC. The Newman strain, α-hemolysin deficient strain, and α-hemolysin complemented strain were kindly provided by Juliane Bubeck Wardenburg (University of Chicago, IL).

### *S. aureus* isolation from human samples

*S. aureus* was collected from the skin lesions of 90 AD patients visiting the Pediatric Clinic of Seoul Suncheonhyang Hospital (Seoul, Republic of Korea). *S. aureus* from healthy controls was isolated from the skin of the upper limbs and subungual spaces from 36 volunteers who had no AD symptoms.

### Isolation of *S. aureus* EVs

*S. aureus* EVs were obtained as described previously [17]. Briefly, *S. aureus* was cultured in nutrient broth or tryptic soybean broth (Difco, Sparks, MD) at 37°C to an optical density (OD) of 1.5 at 600 nm. Bacteria were removed by centrifugation and filtration. The filtrate was concentrated and the resulting concentrate was filtered and ultracentrifuged at 150,000×g for 3 h. The pellet was resuspended in PBS. *S. aureus*-derived EVs protein concentrations were measured using BCA assays (Thermo Scientific, Rockford, IL). Hereafter, the dose of *S. aureus* EVs refers to the quantity of *S. aureus*-derived EVs proteins.

### Cytotoxicity measurements

Keratinocyte viability was measured at 24 h after treatment using thiazolyl blue tetrazolium bromide (MTT) purchased from Sigma Aldrich (St. Louis, MO). The PBS control group was used as 100% viability. Lactate dehydrogenase (LDH) activity in the culture supernatant was measured using the LDH cytotoxicity detection kit purchased from Takara Bio Inc. (Otsu, Japan) according to the manufacturer's instructions.

### Hemolysis measurements

Red blood cells (RBCs) were isolated from mouse whole blood. α-Hemolysin (Sigma-Aldrich, USA) and *S. aureus* EVs were added to RBCs and incubated at 37°C. After 1 h, the remaining RBCs were removed by centrifugation and the optical density at 540 nm of the supernatant was measured. RBC lysis buffer was used as a positive control.

### Flow cytometry analyses of cell death

Annexin V (BD biosciences) and 7-AAD (Biolegend) were used detect cell death using flow cytometry. Cells were treated with *S. aureus* EVs or α-hemolysin, and cells in the culture supernatant and remaining cells were collected. Cells were processed according to the manufacturer's instructions. Processed cells were analyzed using FACSCalibur (Becton Dickinson, USA).

### *In vivo* assays

For Evans blue dye assays and fluorescein-labeled OVA penetration, gauze soaked with 100-µl PBS containing *S. aureus* EVs or α-hemolysin was placed and secured on mildly tape-stripped skin. Mice were treated five times in 1 week with *S. aureus* EVs and α-hemolysin. Dorsal skin was serially fixed with 30, 50, 70, and 100% methanol. After fixation, 0.1% Evans blue was added for 10 min, followed by washing with PBS. Skin was excised, immersed in formamide, and incubated at 60°C. After 6 h, the optical density at 620 nm was measured. For OVA-fluorescein penetration, 50 µg of fluorescein-conjugated OVA were added twice to *S. aureus* EVs- or α-hemolysin-treated skin. Next, the skin was excised and homogenized. Fluorescence was measured using a Wallac 1420 Victor luminometer (American Instrument Exchange, Inc., Harverville, MA). Skin alterations were evaluated after *S. aureus* EVs and α-hemolysin treatment three times per week for 3 weeks, as reported previously [17].

### Statistical analyses

For multiple comparisons, one-way analysis of variance (ANOVA) was used first. If significant differences were found, individual t-tests or Wilcoxon's rank-sum tests were performed between pairs of groups. Differences were considered statistically significant if P<0.05.

## Supporting Information

Figure S1 **A,** Hemolytic activity of EVs and culture media from the ATCC14458 strain and α-hemolysin-deficient EVs strain. Hemolysis mediated by RBC lysis buffer is used as 100%. **B,** The amount of α-hemolysin on EVs from different strains (left panels) and cytotoxicity of EVs on keratinocytes (right panel). ** P<0.01 versus the PBS group.
(EPS)

Figure S2 Quantification of α-hemolysin in EVs. Western blotting showed that 0.6 µg of α-hemolysin was present in 10 µg of EVs protein from the ATCC14458 strain and 0.25 µg of α-hemolysin was present in 10 µg of EVs protein from the Newman strain.
(EPS)

## References

[1] Bieber T (2008) Atopic dermatitis. N Engl J Med 358: 1483–1494. 18385500 10.1056/NEJMra074081

[2] Cork MJ, Danby SG, Vasilopoulos Y, Hadgraft J, Lane ME, et al (2009) Epidermal barrier dysfunction in atopic dermatitis. J Invest Dermatol 129: 1892–1908. 19494826 10.1038/jid.2009.133

[3] Elias PM, Hatano Y, Williams ML (2008) Basis for the barrier abnormality in atopic dermatitis: outside-inside-outside pathogenic mechanisms. J Allergy Clin Immunol 121: 1337–1343. 18329087 10.1016/j.jaci.2008.01.022 PMC2706021

[4] Boguniewicz M, Leung DY (2011) Atopic dermatitis: a disease of altered skin barrier and immune dysregulation. Immunol Rev 242: 233–246. 21682749 10.1111/j.1600-065X.2011.01027.x PMC3122139

[5] Trautmann A, Akdis M, Kleemann D, Altznauer F, Simon HU, et al (2000) T cell-mediated Fas-induced keratinocyte apoptosis plays a key pathogenetic role in eczematous dermatitis. J Clin Invest 106: 25–35. 10880045 10.1172/JCI9199 PMC517909

[6] Rebane A, Zimmermann M, Aab A, Baurecht H, Koreck A, et al (2012) Mechanisms of IFN-gamma-induced apoptosis of human skin keratinocytes in patients with atopic dermatitis. J Allergy Clin Immunol 129: 1297–1306. 22445417 10.1016/j.jaci.2012.02.020

[7] Zimmermann M, Koreck A, Meyer N, Basinski T, Meiler F, et al. (2011) TNF-like weak inducer of apoptosis (TWEAK) and TNF-alpha cooperate in the induction of keratinocyte apoptosis. J Allergy Clin Immunol 127: : 200–207, 207 e201–210. 10.1016/j.jaci.2010.11.005 21211655

[8] Otto M (2010) Basis of virulence in community-associated methicillin-resistant Staphylococcus aureus. Annu Rev Microbiol 64: 143–162. 20825344 10.1146/annurev.micro.112408.134309

[9] Huang JT, Abrams M, Tlougan B, Rademaker A, Paller AS (2009) Treatment of Staphylococcus aureus colonization in atopic dermatitis decreases disease severity. Pediatrics 123: e808–814. 19403473 10.1542/peds.2008-2217

[10] Semic-Jusufagic A, Bachert C, Gevaert P, Holtappels G, Lowe L, et al (2007) Staphylococcus aureus sensitization and allergic disease in early childhood: population-based birth cohort study. J Allergy Clin Immunol 119: 930–936. 17292957 10.1016/j.jaci.2006.12.639

[11] Schlievert PM, Strandberg KL, Lin YC, Peterson ML, Leung DY (2010) Secreted virulence factor comparison between methicillin-resistant and methicillin-sensitive Staphylococcus aureus, and its relevance to atopic dermatitis. J Allergy Clin Immunol 125: 39–49. 20109735 10.1016/j.jaci.2009.10.039 PMC2814367

[12] Bantel H, Sinha B, Domschke W, Peters G, Schulze-Osthoff K, et al (2001) alpha-Toxin is a mediator of Staphylococcus aureus-induced cell death and activates caspases via the intrinsic death pathway independently of death receptor signaling. J Cell Biol 155: 637–648. 11696559 10.1083/jcb.200105081 PMC2198876

[13] Prince LR, Graham KJ, Connolly J, Anwar S, Ridley R, et al (2012) Staphylococcus aureus induces eosinophil cell death mediated by alpha-hemolysin. PLoS One 7: e31506. 22355374 10.1371/journal.pone.0031506 PMC3280314

[14] Walev I, Martin E, Jonas D, Mohamadzadeh M, Muller-Klieser W, et al (1993) Staphylococcal alpha-toxin kills human keratinocytes by permeabilizing the plasma membrane for monovalent ions. Infect Immun 61: 4972–4979. 8225571 10.1128/iai.61.12.4972-4979.1993 PMC281271

[15] Wichmann K, Uter W, Weiss J, Breuer K, Heratizadeh A, et al (2009) Isolation of alpha-toxin-producing Staphylococcus aureus from the skin of highly sensitized adult patients with severe atopic dermatitis. Br J Dermatol 161: 300–305. 19438853 10.1111/j.1365-2133.2009.09229.x

[16] Lee EY, Choi DY, Kim DK, Kim JW, Park JO, et al (2009) Gram-positive bacteria produce membrane vesicles: proteomics-based characterization of Staphylococcus aureus-derived membrane vesicles. Proteomics 9: 5425–5436. 19834908 10.1002/pmic.200900338

[17] Hong SW, Kim MR, Lee EY, Kim JH, Kim YS, et al (2011) Extracellular vesicles derived from Staphylococcus aureus induce atopic dermatitis-like skin inflammation. Allergy 66: 351–359. 20831718 10.1111/j.1398-9995.2010.02483.x PMC3052535

[18] Kulp A, Kuehn MJ (2010) Biological functions and biogenesis of secreted bacterial outer membrane vesicles. Annu Rev Microbiol 64: 163–184. 20825345 10.1146/annurev.micro.091208.073413 PMC3525469

[19] Radjainia M, Hyun JK, Leysath CE, Leppla SH, Mitra AK (2010) Anthrax toxin-neutralizing antibody reconfigures the protective antigen heptamer into a supercomplex. Proc Natl Acad Sci U S A 107: 14070–14074. 20660775 10.1073/pnas.1006473107 PMC2922573

[20] Sato H, Sato Y, Ito A, Ohishi I (1987) Effect of monoclonal antibody to pertussis toxin on toxin activity. Infect Immun 55: 909–915. 2435660 10.1128/iai.55.4.909-915.1987 PMC260437

[21] Bayles KW, Wesson CA, Liou LE, Fox LK, Bohach GA, et al (1998) Intracellular Staphylococcus aureus escapes the endosome and induces apoptosis in epithelial cells. Infect Immun 66: 336–342. 9423876 10.1128/iai.66.1.336-342.1998 PMC107895

[22] Haslinger B, Strangfeld K, Peters G, Schulze-Osthoff K, Sinha B (2003) Staphylococcus aureus alpha-toxin induces apoptosis in peripheral blood mononuclear cells: role of endogenous tumour necrosis factor-alpha and the mitochondrial death pathway. Cell Microbiol 5: 729–741. 12969378 10.1046/j.1462-5822.2003.00317.x

[23] Scaffidi P, Misteli T, Bianchi ME (2002) Release of chromatin protein HMGB1 by necrotic cells triggers inflammation. Nature 418: 191–195. 12110890 10.1038/nature00858

[24] Kim MR, Hong SW, Choi EB, Lee WH, Kim YS, et al (2012) Staphylococcus aureus-derived extracellular vesicles induce neutrophilic pulmonary inflammation via both Th1 and Th17 cell responses. Allergy 67: 1271–1281. 22913540 10.1111/all.12001

[25] O'Regan GM, Sandilands A, McLean WH, Irvine AD (2009) Filaggrin in atopic dermatitis. J Allergy Clin Immunol 124: R2–6. 19720209 10.1016/j.jaci.2009.07.013

[26] Bin L, Kim BE, Brauweiler A, Goleva E, Streib J, et al. (2012) Staphylococcus aureus alpha-toxin modulates skin host response to viral infection. J Allergy Clin Immunol 130: : 683–691 e682. 10.1016/j.jaci.2012.06.019 PMC3594992 22840852

[27] Brauweiler AM, Bin L, Kim BE, Oyoshi MK, Geha RS, et al. (2013) Filaggrin-dependent secretion of sphingomyelinase protects against staphylococcal alpha-toxin-induced keratinocyte death. J Allergy Clin Immunol 131: : 421–427 e421–422. 10.1016/j.jaci.2012.10.030 PMC3742335 23246020

[28] Bomberger JM, Maceachran DP, Coutermarsh BA, Ye S, O'Toole GA, et al (2009) Long-distance delivery of bacterial virulence factors by Pseudomonas aeruginosa outer membrane vesicles. PLoS Pathog 5: e1000382. 19360133 10.1371/journal.ppat.1000382 PMC2661024

[29] Parker H, Chitcholtan K, Hampton MB, Keenan JI (2010) Uptake of Helicobacter pylori outer membrane vesicles by gastric epithelial cells. Infect Immun 78: 5054–5061. 20876296 10.1128/IAI.00299-10 PMC2981328

[30] Kim YR, Kim BU, Kim SY, Kim CM, Na HS, et al (2010) Outer membrane vesicles of Vibrio vulnificus deliver cytolysin-hemolysin VvhA into epithelial cells to induce cytotoxicity. Biochem Biophys Res Commun 399: 607–612. 20682286 10.1016/j.bbrc.2010.07.122

[31] Jin JS, Kwon SO, Moon DC, Gurung M, Lee JH, et al (2011) Acinetobacter baumannii secretes cytotoxic outer membrane protein A via outer membrane vesicles. PLoS One 6: e17027. 21386968 10.1371/journal.pone.0017027 PMC3046175

[32] Gurung M, Moon DC, Choi CW, Lee JH, Bae YC, et al (2011) Staphylococcus aureus produces membrane-derived vesicles that induce host cell death. PLoS One 6: e27958. 22114730 10.1371/journal.pone.0027958 PMC3218073

[33] Thay B, Wai SN, Oscarsson J (2013) Staphylococcus aureus alpha-toxin-dependent induction of host cell death by membrane-derived vesicles. PLoS One 8: e54661. 23382935 10.1371/journal.pone.0054661 PMC3561366

[34] Morfeldt E, Taylor D, von Gabain A, Arvidson S (1995) Activation of alpha-toxin translation in Staphylococcus aureus by the trans-encoded antisense RNA, RNAIII. EMBO J 14: 4569–4577. 7556100 10.1002/j.1460-2075.1995.tb00136.x PMC394549

[35] Ohlsen K, Koller KP, Hacker J (1997) Analysis of expression of the alpha-toxin gene (hla) of Staphylococcus aureus by using a chromosomally encoded hla::lacZ gene fusion. Infect Immun 65: 3606–3614. 9284126 10.1128/iai.65.9.3606-3614.1997 PMC175513

[36] Ohlsen K, Ziebuhr W, Koller KP, Hell W, Wichelhaus TA, et al (1998) Effects of subinhibitory concentrations of antibiotics on alpha-toxin (hla) gene expression of methicillin-sensitive and methicillin-resistant Staphylococcus aureus isolates. Antimicrob Agents Chemother 42: 2817–2823. 9797209 10.1128/aac.42.11.2817 PMC105949

